# Reported Death from Inhalation of Ether

**Published:** 1867-11

**Authors:** 


### Reported Death from Inhalation of Ether.

M. Laroyenne of Lyons, France, reported to the Medical Society of that place the following case: "Subject, a female aged 48 years, constitution feeble. Has an old affection of the left knee, with distortion of the lower extremities. Anæsthesia practiced with caution, insensibility occurring after the inhalation of ten drachms of ether. In two or three minutes, the breathing became embarrassed, face pale, pulse insensible. The recumbent position and cold affusions roused her from this first syncope. Hardly had she been put in bed—fifteen minutes after the first syncope—when a new attack came on, and notwithstanding artificial respiration, prolonged insufflation, galvanization of the diaphragm and the intercostal muscles and of the heart by the aid of long acupuncture needles, it was impossible to call her to life. An attentive examination of the thoracic organs before anæsthesia, had failed to demonstrate any organic lesion. The analysis of the ether by Professor Glenard proved that it contained no foreign substance other than three in 100 parts of water. *Necropsy.* Mucus in the larynx and trachea; pleural adhesions; tubercles in the left pleura; base of left lung congested. The pulmonary tissue was impregnated with the odor of ether. Small quantity of fluid in the pericardium; heart normal; ventricles empty; auricles gorged with blood; nervous centres sound, possessing a feeble odor of ether; a small quantity of fluid in the ventricles. Medulla compressed by a tubercular mass developed in the seventh and eighth dorsal vertebræ, which presented no appearances from without. The alteration had advanced to the left coxofemoral articulation, which presented tubercular masses and osseous fragments within the capsule; head of the femur completely detached; neck totally destroyed. Right hip joint full of blood; recent fracture of the neck of the femur." M. Laroyenne says: "Very evidently the ether was not the sole cause of death in the present case. It acted in favoring the syncope to which the patient was already predisposed by reason of her state of general debility. Assuredly the syncope would not have been produced had the operation been effected without etherization."

